# The Association of Emotional and Physical Reactions to Perceived Discrimination with Depressive Symptoms Among African American Men in the Southeast

**DOI:** 10.3390/ijerph17010322

**Published:** 2020-01-02

**Authors:** Larrell L. Wilkinson, Olivio J. Clay, Anthony C. Hood, Eric P. Plaisance, Lakesha Kinnerson, Brandon D. Beamon, Dominique Hector

**Affiliations:** 1 Department of Human Studies, University of Alabama at Birmingham, Birmingham, AL 35294, USA; plaisep@uab.edu (E.P.P.); lak@uab.edu (L.K.); bbeamon1@uab.edu (B.D.B.); djhector@uab.edu (D.H.); 2 Department of Psychology, University of Alabama at Birmingham, Birmingham, AL 35294, USA; oclay@uab.edu; 3 Department of Management, Information Systems & Quantitative Methods, University of Alabama at Birmingham, Birmingham, AL 35294, USA; anthonychood@uab.edu

**Keywords:** depression, African American, perceived discrimination, adverse reactions, social-emotional support

## Abstract

This study examines the association of emotional and physical reactions to perceived discrimination with depressive symptoms among a sample of African American (AA) men in the southeastern United States. Analysis of the 2010 Behavioral Risk Factor Surveillance System (BRFSS) data set provides an examination of demographic, perceived discrimination context, and health status differences in depressive symptoms measured by the Patient Health Questionnaire—2 (PHQ-2). The analysis also assesses individual differences among AA men related to experiencing physical symptoms and feeling emotionally upset due to perceived discrimination. A focused examination investigates the role of adverse reactions to perceived discrimination in association with depressive symptomology. Findings illuminate the significance of experiences of and reactions to perceived discrimination in relationship with depressive symptomology among AA men living in the southeastern United States. Findings also demonstrate the need for additional research focusing on perceived discrimination experiences in relation to depressive symptoms experienced among the AA male subgroup. Continued investigation of within-group differences among AA men, with health promotional strategies to foster social-emotional support, will further the improvement in health and wellness for AA men.

## 1. Introduction

Mental health is a key component in overall health and well-being. In the United States, nearly one in five adults lives with a mental illness [1]. Serious mental illness (SMI) occurs when individuals experience serious functional impairment substantially interfering with or limiting life activities [1]. Major depressive disorder (or clinical depression) is characterized by two weeks of depressed mood and loss of interest or pleasure (anhedonia) [2]. Depression is predicted to be the second leading cause of global disability burden by 2020 [3]. Among Americans, the annual prevalence of major depressive episodes (MDE) is 17 million, approximately 7 percent of the U.S. adult population, with rates highest among women, young adults, and persons reporting two or more races [4]. The prevalence of MDE among African-Americans (AA) adults was 5.4% in 2017 [4], lower than many sub-groups in the population. Although, previous research indicates AAs are at lesser risk for MDE and other SMIs when compared to European Americans (EA) [5,6], AAs are exposed to a variety of psychosocial stressors contributing to greater risk of MDE [7]. In this vein, perceived discrimination is a stressor associated with deleterious effects on mental health among AA men [8,9,10]. Other factors contributing to depression among AA men are their race and gender, low socioeconomic status, social support status, and physical health status [11]. Underpinning these factors are historical social inequalities drawing from and allowing for the persistence of discrimination within society through group-based hierarchies and societal institutions [12,13,14,15].

Previous studies estimate that 4–12 percent of AA men may experience a major depressive disorder during the previous year [16,17]. Furthermore, findings from the National Survey of American Life: Coping with Stress in the 21st Century (NSAL), revealed 7% of AA men reported lifetime prevalence of Major Depressive Disorder (MDD) [6], while estimates from the National Survey of American Life found 12-month and lifetime prevalence of MDD among AA men to be 5.02% and 9.98%, respectively [18]. Among older AA men, findings from the Chicago Health and Aging Project (CHAP) longitudinal study demonstrated prevalence of MDD to be 11.2% (1993–1997), 20.9% (1997–2000), and 15.9% (2000–2003) during three different time points [19].

The negative impacts of perceived discrimination on mental and physical health have been well documented [8,20,21,22,23,24,25,26,27,28,29,30,31]. Numerous studies show that increased experiences of discrimination are associated with poorer mental health [8,20,22,23,24,28,29,30]. AAs report higher levels of discrimination [30,32,33], and when experiencing perceived discrimination, attribute this to race/ethnicity [30]. AA men report experiences of discrimination at higher levels than women [30,34]. In contrast, AA women report higher rates of psychological distress, depression, anxiety and phobias in comparison to AA men [31,35]; but consequences of adverse mental health such as suicide have increased steadily since the mid-1980s among AA men [36,37].

Studies targeted to examine risk factors within samples of AA men consistently have found perceived discrimination to be predictive of worse outcome measures. Data from 390 AA men included in the 1995 Detroit Area Study (DAS) found that Black men reported more perceived everyday discrimination than did Black women and that discrimination was significantly associated with depressive symptoms, even after controlling for covariates [8]. In another study, 700 AA men were examined to investigate the association between perceived racial discrimination on mental and physical health. In this study, discrimination was significantly associated with worse physical and mental health in AAs, before and after adjustment for age, education, income, and skin color. Specific to mental health, AA men’s mental wellness decreased an average of 0.29 units per unit increase in racial discrimination, becoming 0.32 units after adjustment for age, income, education, and darkness of skin [10]. Other factors related to greater risk of depressive symptomology among AA men include: lower income [18,38] younger age, lower years of education completed, and being separated, divorced, or widowed marital status [18].

In addition to discrimination, previous studies have investigated the associations of socioeconomic position (SEP) and health care with mental health status among AA men. Indeed findings from the Coronary Artery Risk Development in Young Adults (CARDIA) study indicate that among 706 AA men surveyed, 54.0% experienced perceived discrimination at work and 13.5% when accessing medical care [10]. In the CARDIA study, experiencing discrimination at work was associated with worse physical health, while perceiving discrimination in getting medical care was associated with worse mental health [10]. Work stressors [39] and lower employment status (i.e., infrequent work, unemployed), reduced earnings, job instability, and other work-related issues [40] were associated with greater risk for depressive symptomology among adult AA men. Among younger adult AA men, higher job status and job security was associated with lower scoring on a depression scale, indicating more normal mental health [41]. Researchers exploring SEP and depression among AAs, found men with incomes ($80,000 and above) reported greater odds of depression than men in the lowest income category ($17,000 and below) and men who were unemployed [42]. A study of both SEP-based and race-based perceived discrimination on AAs, 22% of whom were men, found 63% perceived discrimination due to their race and 58.9% perceived discrimination due to SEP when encountering health care providers [43].

Perceived discrimination is also linked to experiences of feeling emotionally upset (i.e., angry, sad, or frustrated) and/or feeling negative physical symptoms (i.e., headache, upset stomach, tensing of muscles, or a pounding heart). One study comparing Hispanic/Latinos to Non-Hispanic Whites (NHW) in Arizona found that emotional and physical reactions to perceived discrimination was higher among Spanish-language preferred Hispanic/Latino adults [44]. In addition, as mental and physical health diminished, greater risk for emotional reactions to perceived discrimination was observed [44]. Another study demonstrated linkage between perceived discrimination, psychological distress, and current smoking status using data from the 2004–2008 Behavioral Risk Factor Surveillance System (BRFSS). Within the sample, Non-Hispanic Blacks (NHB) reported discrimination in work and health care environments in greater proportion than NHW or Hispanic/Latino adults. NHBs also reported greater proportions of physical responses to perceived discrimination, and the second greatest proportions of negative emotional responses to perceived discrimination [45]. Employing the BRFSS questionnaire offers a rare opportunity to describe the distribution of emotional and physical reactions to perceived discrimination among AA men, especially in relation to mental health status.

The current study investigates the association of adverse reactions to perceived discrimination experiences with depressive symptomology among AA men in the southeastern United States. In this study, we will identify the prevalence and correlates of experiencing depressive symptomology among AA men in the southeastern United States, while examining the association of experiencing perceived discrimination that results in negative physical symptoms or emotional responses. After determining the relationship between adverse reactions to perceived discrimination and depressive symptomology among AA men in the Southeastern United States, we will determine the magnitude of the relationship and factors that influence the association. Based on the current literature, we hypothesize that greatest risk for depressive symptomology will be among AA men who are exposed to perceived discrimination experiences that lead to negative physical symptoms or emotional responses. We believe risk for depressive symptomology will be greater among AA men who report perceived discrimination experiences at work and in health care settings. Additionally, we theorize having social-emotional support needs unmet may moderate the relationship between adverse reactions to perceived discrimination and depressive symptomology among AA men.

## 2. Materials and Methods

### 2.1. Sample

Data for our analyses came from the 2010 Behavioral Risk Factor Surveillance System (BRFSS)–a population-based telephone survey of health-related behaviors regarding the leading causes of death among noninstitutionalized U.S. adults aged ≥ 18 years. The BRFSS survey design, sampling methods, weights and other details are available at http://www.cdc.gov/brfss/. We analyzed the data collected from survey participants in the state of Georgia, taking advantage of an opportunity to employ both “Anxiety and Depression” and “Reactions to Race” modules, which were implemented in the 2010 BRFSS. During this year, Georgia, Kentucky, & Rhode Island were the only states to administer the “Reactions to Race” module, while Georgia was the only state to administer the “Anxiety and Depression” module among the three states. This rare opportunity to examine the association of emotional and physical reactions to perceived discrimination and depressive symptomology is significant. Additionally, as approximately 55% of AAs live in the southeastern United States [46], investigating this sample of men may enhance understanding of discrimination experiences among AA men.

### 2.2. Measures

Andersen’s behavioral model of health services use supported the framing of this investigation. The model explains how the association of predisposing, enabling, and need factors at the contextual and individual levels, with behavioral factors can influence health outcomes [47,48,49]. Using the model by Andersen [48], we examine predisposing (i.e., gender, age, race/ethnicity), enabling (i.e., income, residence, social-emotional support), and need (i.e., physical health, physical symptoms and/or emotional responses due to perceived discrimination) factors in association with depressive symptomology. Additionally, we investigate processes of medical care (i.e., perceived discrimination in a health care setting) influence on health outcomes (i.e., depressive symptomology).

Dependent Variables-The Patient Health Questionnaire 2 (PHQ-2) was used to assess depressive symptomology status. Specifically, participants were asked two items, over the last two weeks, how many days have you “had little interest or pleasure in doing things?” and “felt down, depressed or hopeless?”. Their responses to each of the questions were categorized as a numerical response between 1–14 days, “none”, “Don’t know/Not sure”, and “Refused”, in appropriation to question format included in other areas of the BRFSS. The BRFSS modified question response set was recoded to the response set used in the Patient Health Questionnaire 9 (PHQ-9), from which the PHQ-2 is derived [50]. The PHQ-2 response set includes: 0 to 1 day = “not at all”, 2 to 6 days = “several days”, 7 to 11 days = “more than half the days,” and 12 to 14 days = “nearly every day”, with points (0 to 3) assigned to each category, respectively [51,52,53]. Participants with a PHQ-2 score of > 3 are indicative of risk for depression [50,54,55]. Thus, responses are dichotomized with scoring > 3 indicating depressive symptomology and normal mental health scoring < 3. Previous studies have demonstrated the validity of the PHQ-2 [50,55].

Independent variables–The main variables of interest in this study include two questions from the “Reactions to Race” module of the BRFSS as factors determining risk for depressive symptomology. Participants were asked, “Within the past 30 days, have you experienced any physical symptoms, for example, a headache, an upset stomach, tensing of your muscles, or a pounding heart, as a result of how you were treated based on your race?” and “Within the past 30 days, have you felt emotionally upset, for example angry, sad, or frustrated, as a result of how you were treated based on your race?” to assess negative physical symptoms and emotional responses to perceived discrimination. Response options were “yes”, “no”, “don’t know/not sure”, and “refused”, with those reporting “refused” being excluded from analysis and “don’t know/not sure” being included with “no” responses.

Subsequently, perceived discrimination at work and in a health care setting was investigated in relationship to depressive symptomology and adverse reactions to perceived discrimination. Perceived discrimination at work was ascertained from participants who reported being employed for wages, self-employed, or unemployed for less than twelve (12) months when asked, “Within the past 12 months at work, do you feel you were treated worse than, the same as, or better than people of other races?” Participants’ responses were categorized as “worse than other races”, “the same as other races”, “better than other races”, “worse than some, better than others”, “only encounter people of the same race”, “don’t know/not sure”, and “refused”. Individuals responding “refused” were excluded from analysis. Additionally, responses regarding perceived discrimination at work were categorized into three separate groups. The first group is “having experienced being treated worse than other races,” combining response selections “worse than other races” with “worse than some, better than others.” The second response group is reporting “no experiences being treated worse than other races,” at work combining response selections “the same as other races”, “better than other races”, “only encounter people of the same race”, and “don’t know/not sure.” Participants who self-reported being unemployed for greater than 1 year, were not asked about their treatment at work, and are included in the “unemployed” category. Finally, participants who reported employment status as “homemaker”, “student”, “retired”, and “unable to work” were formed into one group and included in the sample as well; as this group also was not asked about their treatment at work.

Following, participants perceived discrimination in health care settings was assessed by the item, “Within the past 12 months, when seeking health care, do you feel your experiences were worse than, the same as, or better than for people of other races?”. Participants responses were categorized as “worse than other races”, “the same as other races”, “better than other races”, “worse than some races, better than others”, “only encountered people of the same race”, “no health care in past 12 months”, “don’t know/not sure”, and “refused”. Individuals responding “refused” were excluded from analysis. Perceived discrimination in a health care setting was denoted if participants’ responses included “having experienced being treated worse than other races,” combining response selections “worse than other races” with “worse than some, better than others.” The second response group is reporting “no experiences being treated worse than other races,” at a health care setting combining response selections “the same as other races”, “better than other races”, “only encounter people of the same race”, “no health care in past 12 months” and “don’t know/not sure”.

The demographic variables in our analyses included age (18–39, 40–59, 60+), education (less than high school diploma, high school graduate, and ≥ some college or college graduate), household income (<$25,000, $25,000–50,000, >$50,000), employment status (employed, unemployment experiences during 12 months, Homemaker/Student/Retired/Unable to Work), marital status (currently married, not currently married), and residence (urban, suburban, or rural). Health measures were also included in the analysis. A thirty-day (30) measure in which physical health was not good (0, 1–10, 11–30 days) assessed respondents’ physical health. Perceived social-emotional support was evaluated by asking participants “How often do you get the social/emotional support you need?” Possible responses were “always, usually, sometimes, rarely and never.” To create a dichotomous variable, the responses “sometimes, rarely, and never were grouped into one category labeled as “Social-Emotional Needs Unmet” and the responses “usually and always” as “Social-Emotional Needs Met” [56,57,58].

### 2.3. Analysis Strategy

Weights were applied to the data from the Centers for Disease Control and Prevention to account for survey design, response rates and to yield national (state) estimates for the AA male adult population. Weighted chi-square tests were used to examine the AA male adult population characteristics by mental health status and reactions to perceived discrimination experience. Covariates for inferential analysis were selected based on a review of the scientific literature and variables that were available from the 2010 BRFSS data. To obtain better fit models, covariates were added to the regression models controlling for basic demographic factors (age, education, household income, marital status, residence), discrimination experiences (i.e., at work, in health care settings), and health measures (i.e., physical health, social-emotional support needs met). Weighted logistic regression models generated a crude odds ratio (cOR) estimating the bivariate association of each reaction to perceived discrimination experience and depressive symptoms (Model 1a & 1b). In subsequent multivariate analysis, an adjusted odds ratio (aOR) estimated the association of each reaction to perceived discrimination and depressive symptoms in the presence of significant demographic variables from the Chi-square analysis (Model 2a & 2b). A third model includes significant demographic and discrimination experience variables from the Chi-square analysis (Model 3a & 3b). Finally, a fourth model examines significant demographic and discrimination experience variables from the Chi-square analysis, with physical health and social-emotional support (Model 4a & 4b). For all analyses, survey procedures were used in SAS version 9.4 [59].

## 3. Results

The analysis included three hundred fifty-seven (357) AA men who participated in the BRFSS within the state of Georgia in 2010. According to weighted analysis, an estimated 11.70% of AA men experienced depressive symptoms (depressed mood and anhedonia) during the past 14 days. Table 1 presents the weighted analysis of the demographic variables on depressive symptoms as measured by the PHQ-2. No demographic variables were found to differ significantly by mental health status. Perceived discrimination in a health care setting, experiencing negative physical symptoms as a result of perceived discrimination, experiencing negative emotions as a result of perceived discrimination, and social-emotional support needs being met differed significantly by mental health status. An estimated 18% of AA men with depressive symptoms, experienced perceived discrimination at work (Table 1). Similarly, roughly 20% of AA men with depressive symptoms experienced perceived discrimination in a health care setting. The percentage of AA men with depressive symptoms experiencing negative physical symptoms due to perceived discrimination was 13.53%, with a greater proportion reporting negative emotional responses at 30.83%. Of respondents with depressive symptoms, an estimated 44% reported that their social-emotional support needs were met (Table 1).

An estimated 3.52% of AA men experienced physical symptoms as the result of perceived discrimination over the past 30 days. Significant differences were found among participants regarding education, household income, and days of physical health was not good (Table 2). Among AA men experiencing physical symptoms due to perceived discrimination over the past 30 days, 42% had less than a high school degree (12 years of formal education) and 73% reported household income less than $25,000. In addition, roughly 37% reported 1–10 days of physical health not being good during the past 30 days (Table 2). Although no differences were observed for perceived discrimination experiences at work, perceived discrimination in a health care setting during the past year differed significantly with reporting physical symptoms as the result of perceived discrimination over the past 30 days (*p* = 0.0138). Reporting depressive symptoms during the past 14 days was also associated with physical symptoms due to perceived discrimination (*p* = 0.0015) (Table 2).

An estimated 12.61% of AA men experienced feeling emotionally upset as the result of perceived discrimination during the past 30 days. Significant differences were found among participants by household income and days of physical health was not good (Table 2). Among men with household income less than $25,000, approximately 57% reported feeling emotionally upset as the result of perceived discrimination over the past 30 days. In addition, roughly 36% reported 1–10 days of physical health not being good during the past 30 days (Table 2). Both perceived discrimination experiences at work and perceived discrimination in a health care setting during the past year differed significantly with reporting feeling upset as the result of perceived discrimination over the past 30 days (*p* = <0.0001). Reporting depressive symptoms during the past 14 days was also significantly different by feeling emotionally upset due to perceived discrimination (*p* = 0.0069). Approximately 55% feeling upset due to perceived discrimination, also reported their social-emotional needs being met (Table 2).

In the crude analysis, AA men who experienced negative physical symptoms due to perceived discrimination during the past 30 days, had greater odds of depressive symptoms during the past two weeks than those who reported no physical symptoms due to perceived discrimination (cOR 7.83; 95% CI, 1.81–28.95) (Table 3). In the presence of demographic characteristics, AA men experiencing negative physical symptoms due to perceived discrimination during the past 30 days, had 7 times the odds for depressive symptoms during the past two weeks than those who reported no physical symptoms due to perceived discrimination (Table 3). A significant demographic influencer of the association included less than $25,000 of household income (aOR 3.91; 95% CI, 1.36–11.25) when compared to referent group. The association of experiencing negative symptoms due to perceived discrimination during the past 30 days and reporting depressive symptoms (aOR 6.30; 95% CI, 1.45–27.49) was influenced by reporting less than 25,000 when compared to greater than $50,000 (aOR 3.46; 95% CI, 1.12–10.68) in the presence of experiencing perceived discrimination in a health care setting with education and household income (Table 3). In the full model, having ones social-emotional support needs unmet (aOR 5.81; 95% CI 1.81–18.72) influences the relationship between experiencing negative physical symptoms due to perceived discrimination during the past 30 days and reporting depressive symptoms (aOR 5.75; 95% CI, 1.18–27.94) (Table 3).

Among AA men who had experienced feeling emotionally upset due to perceived discrimination during the past 30 days, there were greater odds of depressive symptoms during the past two weeks than those who reported no physical symptoms due to perceived discrimination (cOR 3.99; 95% CI, 1.37–11.61) (Table 4). When adding household income to the model, men who reported feeling emotionally upset due to perceived discrimination during the past 30 days had greater odds of reporting depressive symptoms (aOR 4.81; 95% CI 1.51–15.29). Although the association between feeling emotionally upset due to perceived discrimination during the past 30 days and reporting depressive symptoms is not significant in Model 3, Model 4 demonstrates that social-emotional support moderates the association, with individuals whose social-emotional support needs being unmet reporting greater risk (aOR 5.95; 95% CI 1.82–19.48) (Table 4).

## 4. Discussion

A sample of 357 AA men in the southeastern United States was examined to assess the association of adverse reactions to perceived discrimination experiences during the past 30 days with depressive symptomology. Furthermore, this association was explored in the presence of geodemographic, perceived discrimination, and health-related characteristics to investigate which factors may influence the relationship between adverse reactions to perceived discrimination experiences during the past 30 days with depressive symptomology. A weighted estimate of 11.70% of AA men reported experiencing depressive symptomology during the past 14 days. In addition, weighted estimates of 3.52% of AA men experienced physical symptoms and 12.61% experienced feeling emotionally upset as the result of perceived discrimination during the past 30 days.

Findings from this study are in accord with earlier research demonstrating the link between perceived discrimination and poorer mental health [8,20,22,23,24,28,29,30]. In contrast to other studies, AA men reporting experiences of being treated worse than other people of a different race at work during the past 12 months did not differ significantly by categorization for depressive symptomology and normal mental health [8,39,40,41,42]. This finding may be due to the inclusion of AA men who were homemakers, students, retirees, unable to work, or reported being unemployed for 1 year or greater. It is plausible that AA men who are students, homemakers, or retirees have less frequent interactions with group-based hierarchies and societal institutions that proliferate discrimination [12,13,14,15], thus reducing their risk for experiences of perceived discrimination during a 12-month period. Consistent with previous studies, risk for depressive symptoms among AA men differed significantly by experiences of perceived discrimination in a health care setting during the past year [8,20,22,23,24,28,29,30].

Other factors did not follow previous literature regarding association with depressive symptomology among this sample of AA men including younger age, lower education status, and reporting marital status as separated, divorce, or widowed [18,38]. Regarding residence, no differences were found in relation to depressive symptoms or adverse reactions to race. Still, reported depressive symptoms among rural AA men may be lower due to the importance of self-reliance, stigma around the concept of depression, and the limited access to mental health services: which may all influence behaviors around mental health among AA men [60].

In this study, experiencing physical symptoms due to perceived discrimination was significantly associated with depressive symptoms. Similarly, reporting feeling emotionally upset in reaction to perceived discrimination was also significantly associated with depressive symptoms. Previous studies have typified the link between experiencing perceived discrimination and mental health outcomes [8,20,22,23,24,28,29,30]. In addition, linkages to reactions to perceived discrimination and the effect on health has been studied [21,44,45]. These findings characterize a physical and emotional response to perceived discrimination, describing the negative impact of perceived discrimination and not just the incidence of the experience. The relationship of these experiences to depressive symptomology is a significant finding, especially among AA men. Regarding the association of experiencing physical symptoms due to perceived discrimination and depressive symptoms, the relationship was influenced by reporting lower levels of household income. A notable finding was the demonstrated influence of unmet social-emotional support need among AA men affecting the association of both physical and emotional reactions to perceived discrimination and depressive symptomology. Unmet social-emotional support needs partially explains the relationship of experiencing physical symptoms due to perceived discrimination with depressive symptomology, reducing the odds from bivariate analysis. Similarly, in the presence of social-emotional support needs being unmet, the association of feeling emotionally upset due to perceived discrimination and depressive symptomology becomes non-significant. Among AA men, these findings affirm the positive role of social support to relieve stress or increase resilience when encountering adverse situations [61].

The results of this study should be interpreted through a perspective of several limitations. The self-report nature of the 2010 administration of the BRFSS is a primary limitation. First, recall bias may limit some of the responses due to solicitation of information for the past 30 days, over the past year, or questions pertaining to ever experiencing frequency of certain conditions or problems during the past 14 days. Secondly, although the PHQ-2 has been used in clinical settings, characterization of its use in population-based surveys is limited. Still, previous studies have used the PHQ-2 in studies with varying sample sizes successfully. Another limitation of this study is the BRFSS 2010 data used in this analysis did not ask questions about mental health knowledge, attitudes, beliefs, or behavioral intentions. This additional information would have been useful in performing data analysis and taking into consideration a factor such as stigma with mental illnesses, mental illness treatment, or social desirability. The limited size of the sample also prevented additional analysis of the data regarding co-factors linked with depressive symptoms. Due to the cross-sectional analysis of the data, the temporal sequence for causation of depressive symptomology among AA men cannot be determined. Future longitudinal work may improve assessment of temporality. Finally, because subgroups of AA men may be homeless or institutionalized, our findings are not generalizable to these subgroups as the BRFSS did not select these groups for interviews.

Despite these limitations, this study is innovative because it takes a population-based approach to examining the mental health status of a defined group that is at risk for poorer mental health due to their high risk for experiencing perceived discrimination. Although not generalizable to the entire U.S. population, or the entire southern United States, one strength of the study is its focus on the AA male population in the southeastern United States. Many studies focus on wide areas of the United States, or AA women within the racial/ethnicity group. This study specifically examines AA male adults. Furthermore, the random selection of the original cohort and the weighting of the data allows this study to make population-based estimates of depressive symptomology with the southern state for which the data is drawn. Finally, the ability to make inferences related to work status, treatment at work, treatment in health care settings, and adverse reactions to perceived discrimination with depressive symptomology among AA men is important as the literature supports the impact each has on AA men’s mental health. Thus, more qualitative efforts are warranted to help supplement quantitative works to enhance understanding of the complex regarding depressive symptomology among AA men and the contextual environment.

## 5. Conclusions

This investigation of depressive symptoms among a sample of AA men adds to the understanding related to the impact of perceived discrimination on mental health. To date, few studies have focused on the AA male population of the United States, and less focusing on the impact of adverse reactions to perceived discrimination, especially in a population with increased risk of discrimination experiences. Future administrations of the BRFSS should encourage more states to adopt the “Reactions to Race” module to study perceived racial discrimination at work and in health care settings, in addition to the adverse reactions to perceived discrimination. This study offered a unique opportunity to estimate the rate of depressive symptoms within this vulnerable sub-group. Additional focused efforts to increase positive social-emotional support and improvements in work and health care environments may prove impactful to advancing better mental health among AA men [62,63,64]. Interventions directly targeting this group may have a significant impact on improving physical and mental health outcomes among AA men, thus impacting their families, and more broadly the AA community.

## Figures and Tables

**Table 1:** Weighted Chi-square analysis of the characteristics of African American (AA) men in the southeastern United States by mental health status, Behavioral Risk Factor Surveillance System (BRFSS) 2010.

Covariates	Depressive Symptoms (*n* = 45)		Normal Mental Health (*n* = 312)		Chi-Square Value	*p*-Value
	*n* ^2^	% ^3^	*n* ^2^	% ^3^	Χ^2^	α = 0.05
Age					0.30	0.8613
18–39	9	44.99	57	39.55		
40–59	23	41.50	154	45.36		
60 and older	13	13.51	101	15.09		
Education					0.20	0.9059
<High School	11	13.13	40	14.47		
=High School	12	29.12	118	32.32		
>High School	22	57.75	154	53.21		
Household Income					5.37	0.0681
<25,000	21	51.13	90	27.60		
25,000–50,000	7	25.39	85	28.15		
>50,000	10	23.48	112	44.25		
Employment Status					1.00	0.6075
Employed	18	61.87	165	65.79		
Un-employment during Past 12 months	9	14.57	23	9.04		
Homemaker/Student/Retired/Unable to Work	18	23.55	124	25.17		
Marital Status					1.47	0.2249
Currently Married	23	51.72	176	65.16		
Not Currently Married	22	48.28	136	34.84		
Place of Residence					2.89	0.2358
Urban	11	36.33	77	22.74		
Suburban	25	52.94	157	56.51		
Rural	9	10.73	78	20.75		
Days Physical Health Not Good					0.66	0.7181
0	18		202			
1–10	12		57			
11–30	13		49			
Perceived Discrimination at Work					3.64	0.3030
Yes	6	18.21	26	8.91		
No	14	47.66	146	60.08		
Homemaker/Student/Retired/Unable to Work	18	23.55	124	25.17		
Unemployed for 1 Year or Greater	7	10.58	16	5.84		
Perceived Discrimination at Health Care Setting					4.56	0.0330 ^1^
Yes	9	19.94	29	7.07		
No	35	80.06	271	92.93		
Negative Physical Symptoms to Perceived Discrimination					10.13	0.0015 ^1^
Yes	5	13.53	10	2.12		
No	40	86.47	290	97.88		
Negative Emotional Responses to Perceived Discrimination					7.30	0.0069 ^1^
Yes	12	30.83	32	10.05		
No	33	69.17	268	89.95		
Social-Emotional Support Needs Met					9.67	0.0019 ^1^
Yes	20	44.44	222	77.48		
No	23	55.56	78	22.52		

^1^ = significant at <0.05, ^2^ = frequency data, ^3^ = weighted data.

**Table 2:** Weighted Chi-square analysis of the characteristics of African American (AA) men in the southeastern United States by experiences of perceived discrimination at work, Behavioral Risk Factor Surveillance System (BRFSS) 2010.

Covariates	Perceived Discrimination Physical Symptoms (*n* = 15)		No Perceived Discrimination Physical Symptoms (*n* = 330)		Chi-Square Measures	Perceived Discrimination Emotional Response (*n* = 44)		No Perceived Discrimination Emotional Response (*n* = 301)		Chi-Square Measures
	*n* ^2^	% ^3^	*n* ^2^	% ^3^	Χ^2^ α = 0.05	*n* ^2^	% ^3^	*n* ^2^	% ^3^	Χ^2^ α = 0.05
Age					0.44 0.8050					3.35 0.1870
18–39	5	49.00	58	40.73		11	55.32	52	38.95	
40–59	6	34.58	165	44.41		27	36.75	144	45.12	
60 and older	4	16.42	107	14.86		6	7.93	105	15.93	
Education					6.15 ^1^ 0.0462					3.65 0.1614
<High School	5	42.05	44	13.7		6	10.22	43	15.20	
=High School	4	26.58	118	29.74		16	45.07	106	27.40	
>High School	6	31.37	168	56.69		22	44.72	152	57.40	
Household Income					15.49 ^1^ 0.0004					11.87 ^1^ 0.0026
<25,000	8	72.89	94	26.21		18	57.07	84	23.60	
25,000–50,000	5	15.27	85	28.18		8	15.01	82	29.60	
>50,000	2	11.84	119	45.61		16	27.93	105	46.80	
Employment Status					4.71 0.0951					0.14 0.9336
Employed	5	34.52	177	69.42		24	66.56	158	68.43	
Un-employment Experience during Past 12 months	2	22.03	27	8.33		4	7.84	25	8.95	
Homemaker/Student/Retired/Unable to Work	8	43.44	126	22.25		16	25.60	118	22.62	
Marital Status					0.62 0.4323					3.59 0.5808
Currently Married	10	76.15	183	63.74		25	46.86	168	66.67	
Not Currently Married	5	23.85	147	36.26		19	53.14	133	33.33	
Place of Residence					0.54 0.7628					1.24 0.5370
Urban	2	26.27	83	24.64		7	22.15	78	25.07	
Suburban	9	45.51	166	56.32		24	49.86	151	56.82	
Rural	4	28.22	81	19.04		13	27.99	72	18.11	
Days Physical Health Not Good					6.52 ^1^ 0.0384					6.15 ^1^ 0.0462
0	3	34.14	213	71.74		20	54.76	196	72.59	
1–10	4	37.32	61	18.08		12	35.57	53	16.41	
11–30	8	28.54	50	10.18		10	9.67	48	11.00	
Perceived Discrimination at Work					-					46.27 ^1^ <0.0001
Yes	5	34.52	27	9.62		17	45.42	15	5.46	
No	0	-	159	63.37		9	27.39	150	66.01	
Not Asked	8	43.44	126	22.25		16	25.60	118	22.62	
Unemployed for 1 Year or Greater	2	22.03	18	4.76		2	1.59	18	5.91	
Perceived Discrimination at Health Care Setting					6.06 ^1^ 0.0138					47.20 ^1^ <0.0001
Yes	6	29.09	32	7.89		17	41.36	21	3.91	
No	9	70.91	297	92.11		27	58.64	279	96.01	
Depressive Symptoms					10.13 ^1^ 0.0015					7.30 ^1^ 0.0069
Yes	5	47.22	40	11.01		12	30.05	33	9.72	
No	10	52.78	290	88.99		32	69.95	268	90.28	
Social-Emotional Support Needs Met					2.96 0.0852					4.14 ^1^ 0.0420
Yes	8	46.08	224	73.45		28	54.78	204	75.18	
No	6	53.92	93	26.55		16	45.22	83	24.82	

**^1^** = significant at <0.05, ^2^ = frequency data, ^3^ = weighted data.

**Table 3:** Crude and adjusted associations between physical symptoms from perceived discrimination and self-reported depressive symptoms among African American (AA) men in the Southeastern United States, 2010.

Covariates	Model 1 Depressive Symptoms	Model 2 Depressive Symptoms	Model 3 Depressive Symptoms	Model 4 Depressive Symptoms
	cOR (95% CI)	aOR (95% CI)	aOR (95% CI)	aOR (95% CI)
Perceived Discrimination (Physical Symptoms)				
Physical Symptoms from Perceived Discrimination vs. No Reported Physical Symptoms	7.23 ^1^ (1.81–28.95)	7.13 ^1^ (1.78–28.63)	6.30 ^1^ (1.45–27.49)	5.75 ^1^ (1.18–27.94)
Education				
=High School vs. >High School	-	0.78 (0.26–2.34)	0.76 (0.23–2.46)	0.93 (0.31–2.75)
<High School vs. >High School	-	0.50 (0.16–1.55)	0.55 (0.18–1.69)	0.63 (0.17–2.37)
Household Income				
<25,000 vs. >50,000	-	3.91 ^1^ (1.36–11.25)	3.46 ^1^ (1.12–10.68)	3.41 (0.88–13.16)
25,000–50,000 vs. >50,000	-	1.96 (0.49–7.80)	1.86 (0.50–6.96)	1.20 (0.26–5.51)
Perceived Discrimination at Health Care Setting				
Yes vs. No			2.26 (0.44–11.52)	1.56 (0.27–9.04)
Physical Health Days Not Good				
1–10 Days vs. 0 Days				0.58 (0.08–1.55)
11–30 Days vs. 0 Days				
Social-Emotional Support Needs Met				
No vs. Yes				5.81 ^1^ (1.81–18.72)

Abbreviations: OR, Odds Ratio; cOR, Crude OR; aOR, Adjusted OR; CI, Confidence Interval. Fully adjusted model included physical symptoms due to perceived discrimination, education, the household income, perceived discrimination experience in health care setting, physical health days not good, and social-emotional needs met. ^1^ CI does contain not 1.00.

**Table 4:** Crude and adjusted associations between emotional responses to perceived discrimination and self-reported depressive symptoms among African American (AA) men in the Southeastern United States, BRFSS 2010.

Covariates	Model 1 Depressive Symptoms	Model 2 Depressive Symptoms	Model 3 Depressive Symptoms	Model 4 Depressive Symptoms
	cOR (95% CI)	aOR (95% CI)	aOR (95% CI)	aOR (95% CI)
Perceived Discrimination (Negative Emotional Responses)				
Negative Emotional Responses from Perceived Discrimination vs. No Reported Emotional Responses	3.99 ^1^ (1.37–11.61)	4.81 ^1^ (1.51–15.29)	4.12 (0.97–17.53)	3.57 (0.96–13.27)
Household Income				
<25,000 vs. >50,000		2.89 (0.96–8.67)	2.80 (0.79–9.98)	3.08 (0.64–14.84)
25,000–50,000 vs. >50,000		1.90 (0.54–6.74)	1.79 (0.51–6.30)	1.11 (0.25–5.00)
Perceived Discrimination at Work				
Yes vs. No			1.29 (0.29–5.81)	1.45 (0.37–5.78)
Homemaker/Student/Retired/Unable to Work vs. No			0.98 (0.26–3.67)	0.61 (0.12–3.21)
Unemployed for one year or greater vs. No			1.49 (0.27–8.13)	0.61 (0.11–3.51)
Perceived Discrimination in Health Care Setting				
Yes vs. No			1.26 (0.26–5.96)	0.88 (0.24–3.32)
Physical Health Days Not Good				
1–10 Days vs. 0 Days				0.60 (0.17–2.13)
11–30 Days vs. 0 Days				0.73 (0.20–2.68)
Social-Emotional Support Needs Met				
No vs. Yes				5.95 ^1^ (1.82–19.48)

Abbreviations: OR, Odds Ratio; cOR, Crude OR; aOR, Adjusted OR; CI, Confidence Interval; Fully adjusted model included the household income, perceived discrimination at work, perceived discrimination at a health care setting, physical health days not so good, and social-emotional needs met. ^1^ CI does not contain 1.00.
